# Volunteer Service and Well-Being of Older People in China

**DOI:** 10.3389/fpubh.2022.777178

**Published:** 2022-02-25

**Authors:** Hua-lei Yang, Shuo Zhang, Wen-chao Zhang, Zheng Shen, Jia-hao Wang, Si-meng Cheng, Yi-wen Tao, Si-qing Zhang, Li-xingzi Yang, Yi-dan Yao, Lin Xie, Li-li Tang, Yuan-yang Wu, Zhi-yun Li

**Affiliations:** ^1^School of Public Administration, Zhongnan University of Economics and Law, Wuhan, China; ^2^School of Economics and Management, Zhejiang A&F University, Hangzhou, China; ^3^Institution of Population and Labor Economics, University of Chinese Academy of Social Science, Beijing, China; ^4^School of Chemistry and Chemical Engineering, Yantai University, Yantai, China; ^5^College of Politics and Public Administration, Qingdao University, Qingdao, China

**Keywords:** older people, volunteer service, well-being, propensity score match, positive emotion

## Abstract

**Purpose:**

The social support theory suggested that involving older people in social activities could increase their level of social participation and interaction, which in turn improved their well-being. However, there has been a heated controversy about whether participating in volunteer services could enhance the well-being of older people, especially for the Chinese sample.

**Method:**

Based on the data from the China Health and Retirement Longitudinal Study (CHARLS) in 2013, this paper used an ordered probit model to examine the impact of older people's participation in volunteer services on their well-being, as well as the differences in the impact across groups and the specific transmission mechanism.

**Result:**

The empirical study found that Chinese older people's participation in volunteerism significantly enhanced their well-being, which remained robust after eliminating the possible effects of self-selection. Further heterogeneity analysis revealed that for female, non-party members and older adults with good economic status, participation in volunteerism has a higher increase in well-being. The mediating effect test indicated that older people's participation in volunteerism affected well-being mainly through enhancing positive emotions.

**Conclusion:**

It is necessary to promote the participation of older people in volunteer services and to clarify the role of government support and advocacy. Proper guidance is given to change the role of older people as care recipients to that of service providers and caregivers, and to continuously enrich the programmes and content of volunteer service to safeguard the well-being of older people.

## Introduction

At present, the population aging process of China is accelerating, and the number of the elderly population is increasing. Data from the seventh National Census in 2020 showed that China's elderly population aged 60 and above has reached 264 million, accounting for about 18.70% of the total population. By 2050, the elderly population is expected to reach 8.7 billion, accounting for 34.9% of the total population (1). In this demographic context, the mental health of older people has been drawing widespread attention from the whole society. The *China Health and Retirement Report* stated that about 33.1% of the Chinese population over the age of 60 was at high risk of depression (2). Based on a detailed analysis of data from the 2014 China Longitudinal Aging Social Survey (CLASS), ~24.78% of Chinese older people experience varying degrees of loneliness (3). Social participation theory suggests that social participation can help older people re-establish social networks, find social roles, recognize themselves in their roles, and enhance their sense of well-being. Supportive social networks can enable people to engage and stay involved in volunteering by providing instrumental and emotional support (4). It is widely accepted that participation in volunteering is associated with higher life satisfaction (5, 6), happiness (5) and positive emotion (6, 7) are related. At the same time, volunteer service may also promote the formation of supportive social relationships. There is broad consensus that supportive social relationships are beneficial to mental health and well-being (8, 9), by promoting positive emotions and acting as a stress buffer (10, 11).

According to the *Charity Blue Book: China Charity Development Report* in 2020, the number of registered volunteers in China reach 169 million, the total number of volunteer groups reach 1,163,600, and the accumulated volunteer service time reach 2,268 million hours. Specifically, among those who participate in volunteer services, the percentage of CPC (Communist Party of China) members is 13.81%. In addition, residents with higher level of education are more likely to volunteer, with the highest percentage of active volunteers with college education and above. However, there are still some shortcomings in China's current volunteer activities. Less than 10% of older volunteers over the age of 60 (12), and the main type of older people's volunteer service is informal volunteering, which refers to unpaid assistance to relatives, friends or neighbors who do not live together, and the participation of older people is obviously insufficient. In addition, there are differences in the participation rates of various types of volunteer service in China in terms of identity, generation, region, urban and rural areas, and the demand for volunteer service is much higher than the current actual participation rate. Therefore, how to further promote the development of volunteerism in China and encourage more people to participate has become a key concern for policy makers as well as many scholars.

Happiness is an important indicator of the quality of life of older people. In the modern sense, starting with the *Factors associated with self-reported happiness* of Wilson published in 1967, it refers to a series of emotions of delight and pleasure that humans subjectively produce based on their own satisfaction and security. This paper does not make a strict distinction between the concepts of happiness, subjective well-being, and life satisfaction. Therefore, based on the backgrounds above, this paper puts forward the topic to be discussed, that is, whether the participation of the Chinese older people in volunteer services will help to improve their well-being in order to deal with the happiness crisis? Besides, if there is a positive connection between the two, is there a difference in this connection among different groups? Then what is its mechanism?

Scholars all over the world have carried out different degrees of discussion on the impact of volunteer service on older people's well-being. This paper combs the impact results, impact extents and impact mechanism.

For the impact result, most scholars have found that volunteer service helps to improve the well-being of older people. Based on the survey data of life change in the United States, Willigen (13) used OLS model found that the more older people participated in volunteer services, the higher their happiness, while this impact was greater for older people than for younger groups. Meanwhile, Haski-Leventhal (14), Andiara et al. (15), Sarid et al. (16) and Ho (17) also used the survey data of Europe, Singapore, Israel and Pingtung County in Taiwan to verify the positive impact of volunteer service on the well-being. However, some scholars have found that there may be no connection between the two. For example, Menec (18) operationalised well-being as happiness and life satisfaction based on Manitoba aging survey data, and it was found that there was no significant relationship between volunteer service and happiness or life satisfaction. In a study of older people in Israel, Shmotkin et al. (19) also found that while volunteering had a significant effect on self-rated health and social relations, it did not have a significant effect on current life evaluation.

In terms of the degree of impact, there are differences in the promotion of well-being by older people participating in volunteer services. Firstly, different participation frequencies have different degrees of influence. Morrow-Howell et al. (20) found that there was a non-linear relationship between the frequency of volunteer service participation and well-being, which meant that there was an optimal frequency of participation. Windsor et al. (6) pointed out that there was an inverted U-shaped relationship between volunteer service and well-being. Older people with high levels of involvement had higher level of negative emotions than those who were not involved and those who were moderately involved in volunteering. However, Christina et al. (21) noted that older people with low and moderate levels of involvement had lower psychological well-being than those without volunteering, while only high levels of involvement significantly increased the psychological well-being of them. Secondly, Changes in the status of participation in volunteer services can also lead to changes in impact effects. Li et al. (22) used the data of the Taiwan older people's health and life survey, dividing the volunteer service status into five states, and it was found that “continuous participation” and “from inactive to active participation” have a significant positive impact on life satisfaction and depression, while “discrete participation” and “from active to inactive participation” only have a significant positive impact on life satisfaction. Thirdly, the degree of influence will vary depending on the characteristics of the individuals involved in the volunteer services. Connolly and O' Shea (23) found that the impact of volunteer service was greater for older, less educated, and retired groups. Besides, based on 2005 CGSS data, Miao and Zhang (24) used a treatment effects model and found that there was a social compensation effect when older people in disadvantaged positions participated in volunteering. In other words, disadvantaged older people were better able to enhance their well-being through volunteering. Additionally, Jiang et al. (25) found that different social networks had different impact effects. For older people with fewer friends, the impact of volunteer service was more obvious.

As to the influence mechanism, scholars put forward different views based on different theoretical frameworks. By operationalising subjective well-being into life satisfaction, positive emotions and negative emotions, Greenfield and Marks (7) found that older people's participation in volunteering had a significant effect on life satisfaction as well as positive emotions, while the effect on negative emotions was not significant. In this regard, they believed that the impact of volunteer service on well-being was mainly achieved by improving life satisfaction and positive emotions. Similarly, Kahana et al. (26) reached the same conclusion. In addition, based on the Wisconsin follow-up survey data, Piliavin and Siegl (27) pointed out that older people's participation in volunteer services affected their mental health through social integration and the sense of being valued. The former meant that older people with less social relations were more likely to improve their mental health through volunteer service, while the latter meant that older people can realize their importance through volunteer service, which affected their mental health. From a social support perspective, Pilkington et al. (4) hypothesized that the impact of older people's volunteering on well-being was mainly through the type and quality of social support, and their findings suggested that social support from friends and family and positive social exchange played a mediating role.

Through the above literature review, it is found that participating in volunteer services may improve well-being of older people by improving their positive emotions, self-identity, and social support, while the frequency of individual involvement, changes in service status and differences in individual characteristics will in turn lead to differences in the degree of impact. These research conclusions are mainly based on the actual situation of developed countries. As a developing country, China's social development and the living situation of older people may have particularity, so the impact of volunteer service on well-being of them may be different. At the same time, considering the low proportion of older people participating in volunteer services, the single content of activities and the mandatory and obligatory characteristics of voluntary service, it is also worth exploring whether the above mechanisms of volunteer service on the well-being of older people are suitable for China. The existing research results provide a variety of possibilities. What is the relationship and mechanism between volunteer service and well-being of older people in China? We explored this in depth.

## Method

### Data

The data of this paper was from the 2013 China Health and Retirement Longitudinal Study (CHARLS), which was a large-scale interdisciplinary survey project hosted by the National Development Research Institute of Peking University. It aimed to collect a set of high-quality microdata representing the families and individuals of middle-aged and elderly people aged 45 and over in China. Referring to the *Law of the People's Republic of China on the Protection of the Rights and Interests of the Elderly*, this paper selected samples aged 60 and above and retained 4,403 samples after screening variable and cleaning data.

### Variable

#### Dependent Variable

The dependent variable was well-being, which was people's evaluation of their own life, including emotional and cognitive aspects (28). Ma (29) pointed out that life satisfaction was a stable measure of people's long-term well-being based on a comprehensive evaluation of the multidimensional aspects of life and only the indicator of life satisfaction was available in the 2013 questionnaire, so this paper used life satisfaction to represent subjective well-being. Based on the question of “Overall, are you satisfied with your life?,” well-being was divided into five levels.

#### Independent Variable

The independent variable was volunteer service which was defined as public service provided by volunteers, voluntary service organizations, and other organizations to society or others voluntarily and without compensation in the *Voluntary Service Ordinance*. Considering that volunteering in China was characterized by unpaid service and helping people (30), this paper regarded “providing free help to relatives, friends or neighbors who do not live with you” as informal volunteering, and “volunteering or charitable activities” as formal volunteering. Respondents who participated in at least one of them were defined as participating in volunteering.

#### Control Variable

Considering that other factors had an impact on well-being, this paper selected the respondents' individual, family, and socio-economic characteristics as control variables referring to the research of Wang (31). The details were shown in Table 1.

**Table 1:** Variable definition.

**Variable**	**Variable definition**
Happiness (*happiness)*	Not satisfied at all = 1, not very satisfied = 2, relatively satisfied = 3, very satisfied = 4, extremely satisfied = 5
Volunteer service (*volunteer)*	Participating in voluntary service = 1, not participating in voluntary service = 0
Gender (*gender)*	Male = 1, female = 0
Age (*age)*	2013-year of birth
ADL (*adl)*	Activities of Daily Living, whether there were difficulties in dressing, eating, bathing, getting in and out of bed, going to the toilet, and controlling bowel movements. Continuous variable. The higher the value, the greater the difficulty.
IADL (*iadl)*	Instrumental Activity of Daily Living, whether there were difficulties in doing housework, cooking, shopping, making phone calls, taking medicine, and managing money. Continuous variable. The larger the value, the greater the difficulty.
Chronic disease (*cdisease)*	Number of chronic diseases
Partner (*partner)*	With partner =1, no partner =0
Live with children (*livechild)*	Yes = 1, no = 0
Number of family members (*hhsize)*	Number of family members
Children's financial support (*lnfinsup)*	The sum of money and material provided by children in the past year, and taking the logarithm.
political look (*party)*	Chinese Communist Party member = 1, others = 0
Years of education (*edu)*	Illiterate = 0, unfinished primary school or private school = 3, primary school graduation = 6, junior middle school graduation = 9, high school or technical secondary school graduation = 12, college graduation = 15, undergraduate graduation = 16, master graduation = 19
Pension insurance (*pension)*	Having pension insurance = 1, not having pension insurance = 0
City (*urban)*	Urban = 1, rural = 0
Working status (*work)*	With work =1, no work = 0
Economic status (*lnasset)*	The sum of personal financial assets (cash, deposits of financial institutions, bonds, stocks, and funds), and taking the logarithm.
Positive emotion (*positive)*	The higher the value, the more positive.
Negative emotion (*negative)*	The higher the value, the more negative.

*The name of variable in the model were indicated in parentheses*.

#### Mediating Variable

Liu (32) stated that social interaction affected older people's life satisfaction through depression. Meanwhile, Greenfield and Marks (7) found that older people's involvement in volunteering could enhance well-being through positive emotions. In order to investigate the mechanism, the responses to “I'm worried about some small things,” “I feel depressed,” “I feel scared,” “I feel lonely,” and “I feel unable to continue with my life' were assigned a value of 0–3 in turn, and summed to obtain the level of negative emotion. Similarly, positive emotion level was obtained through ‘I am hopeful about the future” and “I am happy.”

### Model

#### Ordered Probit Model

As the dependent variable was an ordered variable, this paper used an ordered probit model for estimation, which was set up as followed:


(1) $$\begin{array}{l} happeniness_{i}=F\left(c*volunteer_{i}+\gamma X_{i}+\varepsilon_{i}\right) \end{array}$$

*volunteer*_*i*_ indicated participation in volunteer service; *X*_*i*_ was a series of control variables; ε_*i*_ was the random error term. In addition, referring to Chen et al. (33) who regarded discrete variable as ordinal variable and then adopted OLS regression model, this paper also added OLS regression results to compare with the results of ordered probit model.

#### Propensity Score Matching

The participation of older people in voluntary service was a self-selection behavior (24, 34). For example, happier people are more likely to participate in voluntary service. If it was not handled effectively, the impact of participation in voluntary services on well-being in the regression results may not have internal validity. PSM could well-eliminate the endogenous threat caused by self-selection. The participants' average treatment effect (ATT) was obtained through the following model:


(2) $$\begin{array}{l} ATT=E\left[happeniness_{1i}|D_{i}=1,p\left(X_{i}\right)\right]\\ \;-E\left[happeniness_{0i}|D_{i}=0,p\left(X_{i}\right)\right] \end{array}$$

Here, *happeniness*_1*i*_ was the well-being of treatment group; *happeniness*_0*i*_ was the well-being of control group. *D*_*i*_ = 1 indicated participation in volunteer services, while*D*_*i*_ = 0 indicated that older people did not participate in volunteer services.

#### Mediating Effect Test

In order to explore whether participation in voluntary services affected well-being by improving positive emotions and inhibiting negative emotions, this paper added formulas (3) and (4) referring to Wen and Ye (35):


$$\begin{array}{l} happeniness_{i}=F\left(c*volunteer_{i}+\gamma X_{i}+\varepsilon_{i}\right)\left(1\right)\\ \;M_{i}=a*volunteer_{i}+\gamma X_{i}+\varepsilon_{i}\left(3\right)\\ happeniness_{i}=F\left(c^{\prime }*volunteer_{i}+b*M_{i}+\gamma X_{i}+\varepsilon_{i}\right)\left(4\right) \end{array}$$

Here, *c* represented the total effect of participation in voluntary services on well-being. *a* was the effect of voluntary services on intermediary variable *M*_*i*_. *b* was the effect of intermediary variable on well-being. *c*′ represented the direct effect of voluntary service on well-being under the control of intermediary variable. At this time, the intermediary effect was equal to the indirect effect, expressed as a coefficient product *ab*.

## Results

### Statistical Description of Variables

Table 2 showed the main statistics of variables. As shown in Table 2, the mean value of well-being level was 3.151, which was generally at a relatively satisfactory level. Among them, the well-being level of older people who participated in volunteering was 3.262, higher than those who did not, and this difference was statistically significant at the 1% level. In terms of volunteer service participation, about 10.2% of the sample participated in volunteer services, indicating that the current level of volunteering participation for Chinese older people was low.

**Table 2:** Descriptive statistics of variables.

**Variable**	**Total**				**Participating in**		**Not participating in**	
	**(*****N*** **=** **4,403)**				**volunteer service**		**volunteer service**	
					**(*****N*** **=** **450)**		**(*****N*** **=** **3,953)**	
	**Mean**	**Std.Dev**	**Min**	**Max**	**Mean**	**Std.Dev**	**Mean**	**Std.Dev**
Happiness	3.151	0.744	1	5	3.262***	0.754	3.138	0.742
Volunteer	0.102	0.303	0	1	–	–	–	–
Gender	0.464	0.499	0	1	0.480	0.500	0.462	0.499
Age	67.958	6.346	60	95	66.231***	5.324	68.155	6.423
Cdisease	1.529	1.399	0	8	1.578	1.442	1.523	1.394
Adl	0.712	1.688	0	17	0.602	1.308	0.724	1.726
Iadl	1.724	3.178	0	18	1.087***	2.104	1.796	3.271
Partner	0.819	0.385	0	1	0.822	0.383	0.818	0.386
Hhsize	4.648	1.733	1	15	4.722	1.806	4.64	1.724
Livechild	0.471	0.499	0	1	0.500	0.501	0.468	0.499
Lnfinsup	6.596	2.953	0	12.567	6.709	2.814	6.584	2.968
Party	0.126	0.332	0	1	0.149	0.356	0.124	0.329
Edu	4.094	3.830	0	19	4.936***	4.076	3.998	3.790
Pension	0.903	0.295	0	1	0.904	0.294	0.903	0.295
Work	0.531	0.499	0	1	0.627***	0.484	0.520	0.500
Lnasset	5.985	3.119	0	13.999	6.508***	3.213	5.926	3.102
Urban	0.377	0.485	0	1	0.400	0.490	0.375	0.484
Negative	2.814	3.332	0	15	2.807	3.133	2.815	3.354
Positive	2.779	2.08	0	6	3.211***	2.052	2.730	2.078

***, ** and *, respectively, indicate the significance of the t-test results of the difference between the volunteer service group and the non-volunteer service group at the statistical level of 1, 5, and 10%.

### Basic Regression Results

Table 3 showed the regression results, in which models (1) and (2) were the ordered probit regression results, and models (3) and (4) were the OLS regression results. According to the results of both models, we could know that the participation of older people in voluntary service has a positive impact on their well-being. That is, older people who participated in volunteer services had an increased sense of well-being compared to those who did not, which was significant at the 1% level. This was in line with the research of other scholars (4, 13).

**Table 3:** Basic regression results.

	**Ordered Probit**		**OLS**	
	**(1)**	**(2)**	**(3)**	**(4)**
Volunteer	0.179***	0.207***	0.124***	0.139***
	(0.055)	(0.056)	(0.037)	(0.037)
Gender		−0.012		−0.003
		(0.037)		(0.025)
Age		0.019***		0.013***
		(0.003)		(0.002)
Cdisease		−0.044***		−0.029***
		(0.012)		(0.008)
Adl		−0.043***		−0.030***
		(0.012)		(0.008)
Iadl		−0.018***		−0.013***
		(0.007)		(0.005)
Partner		0.120**		0.075**
		(0.051)		(0.034)
Hhsize		−0.000		0.001
		(0.012)		(0.008)
Livechild		0.077**		0.049**
		(0.038)		(0.025)
Lnfinsup		0.025***		0.016***
		(0.006)		(0.004)
Party		0.048		0.031
		(0.054)		(0.035)
Edu		−0.021***		−0.014***
		(0.005)		(0.003)
Pension		0.121**		0.085**
		(0.057)		(0.038)
Work		−0.033		−0.024
		(0.039)		(0.026)
Lnasset		0.019***		0.013***
		(0.006)		(0.004)
Urban		0.113***		0.071***
		(0.039)		(0.025)
Cons			3.138***	2.054***
			(0.012)	(0.156)
*N*	4,403	4,403	4,403	4,403
*R* ^2^			0.003	0.042

These in brackets are standard errors; ***, ** and * indicate the significance at the statistical level of 1, 5, and 10%, respectively.

In terms of control variables, age (36), social pension insurance, financial situation, and town life had a significant effect on subjective well-being. Spouse status, living with children and children's economic support also could significantly promoted their well-being (37). Limited ability to perform daily activities and chronic illnesses could reduce the well-being. It was surprising that there was a significant negative relationship between education level and subjective well-being (33).

### Robustness Test

In order to eliminate the selection bias caused by self-selection, propensity score matching method was used for robustness test in this paper. A balance test was conducted and the results were shown in Table 4. The deviation rate of all variables after matching was reduced to <2.5%, and the difference between the treatment and the control group was not significant, which also showed that the matching effect was good.

**Table 4:** Balance test.

**Variable**	**Mean value**				**Deviation reduction rate (%)**	***T*** **-test**	
	**Sample**	**Processing group**	**Control group**	**Deviation rate (%)**		***T*-score**	**P>|T|**
Gender	Before matching	0.480	0.462	3.6	98.8	0.72	0.473
	After matching	0.480	0.480	0.0		0.01	0.995
Age	Before matching	66.231	68.155	−32.6	97.1	−6.12	0.000
	After matching	66.231	66.176	0.9		0.15	0.877
Cdisease	Before matching	1.578	1.523	3.9	5.2	0.79	0.432
	After matching	1.578	1.526	3.7		0.54	0.586
Adl	Before matching	0.602	0.724	−8.0	78.7	−1.45	0.147
	After matching	0.602	0.576	1.7		0.29	0.776
Iadl	Before matching	1.087	1.796	−25.8	90.2	−4.50	0.000
	After matching	1.087	1.017	2.5		0.48	0.630
Partner	Before matching	0.822	0.818	1.0	36.6	0.20	0.841
	After matching	0.822	0.825	−0.6		−0.10	0.923
Hhsize	Before matching	4.722	4.640	4.7	69.2	0.95	0.340
	After matching	4.722	4.748	−1.4		−0.21	0.832
Livechild	Before matching	0.500	0.468	4.3	77.0	0.85	0.394
	After matching	0.500	0.506	−1.0		−0.15	0.879
Lnfinsup	Before matching	6.709	6.584	4.3	77.0	0.85	0.394
	After matching	6.709	6.738	−1.0		−0.15	0.879
Party	Before matching	0.149	0.124	7.3	98.2	1.52	0.128
	After matching	0.149	0.148	0.1		0.02	0.985
Edu	Before matching	4.936	3.999	23.8	92.5	4.93	0.000
	After matching	4.936	4.865	1.8		0.27	0.791
Pension	Before matching	0.904	0.903	0.4	−85.2	0.07	0.941
	After matching	0.904	0.906	−0.7		−0.10	0.918
Work	Before matching	0.627	0.520	21.6	93.9	4.29	0.000
	After matching	0.627	0.633	−1.3		−0.20	0.842
Lnasset	Before matching	6.508	5.926	18.4	87.0	3.75	0.000
	After matching	6.508	6.432	2.4		0.37	0.713
Urban	Before matching	0.400	0.375	5.2	60.5	1.05	0.293
	After matching	0.400	0.390	2.1		0.31	0.759

This paper used K-nearest neighbor matching, radius matching and kernel matching methods to match the treatment group with the control group, and then calculated the treatment effect. The results in Table 5 showed that participation in volunteer services by older people was an effective way to improve well-being. By comparing the pre-matching and post-matching results, it could be found that all post-matching results were improved, suggesting that the effect of older people's participation in volunteering on well-being was underestimated when the effect of self-selection waas not taken into account.

**Table 5:** Average processing effect of participation in volunteer services on well–being.

**Method**	**Sample**	**Processing group**	**Control group**	**ATT**	**Standard error**
K-nearest neighbor matching	Before matching	3.262	3.138	0.124***	0.037
	After matching	3.262	3.117	0.145***	0.041
Radius matching	Before matching	3.262	3.138	0.124***	0.037
	After matching	3.262	3.127	0.133***	0.038
Kernel matching	Before matching	3.262	3.138	0.124***	0.037
	After matching	3.262	3.127	0.136***	0.038

***, ** and *, respectively, indicate the significance of variables at the statistical level of 1, 5, and 10%; The matched standard error is obtained by bootstrap method, and the self-service sample is 500.

### Heterogeneity Analysis

In order to further discuss the differences in the impact of older people's participation in volunteer services on their well-being, this paper used PSM method to test gender, political look and economic status, respectively. The results were shown in Table 6.

**Table 6:** Heterogeneity test results.

**Method**	**Male**			**Female**		
	***N***	**ATT**	**Standard error**	***N***	**ATT**	**Standard error**
K-nearest neighbor matching	2,031	0.115*	0.062	2,335	0.141**	0.056
Radius matching	2,028	0.114**	0.055	2,328	0.143***	0.054
Kernel matching	2,031	0.116**	0.055	2,335	0.156***	0.050
**Method**	**Party member**			**Non-party member**		
	***N***	**ATT**	**Standard error**	***N***	**ATT**	**Standard error**
K-nearest neighbor matching	516	0.006	0.093	3,838	0.158***	0.043
Radius matching	508	0.028	0.099	3,836	0.148***	0.042
Kernel matching	516	0.031	0.092	3,838	0.152***	0.043
**Method**	**Good**			**General**		
	***N***	**ATT**	**Standard error**	***N***	**ATT**	**Standard error**
K-nearest neighbor matching	2,451	0.135**	0.054	1,916	0.115	0.076
Radius matching	2,451	0.134***	0.045	1,912	0.116*	0.067
Kernel matching	2,451	0.128***	0.045	1,916	0.129*	0.068

***, ** and *, respectively, indicate the significance of variables at the statistical level of 1, 5, and 10%; The matched standard error bootstrap shows that there are 500 self-service samples; The sample size here is the sample in the common support interval.

From the perspective of gender, participation in volunteer services significantly increased the well-being of both male and female older people. In particular, participation in volunteer service contributed more to the well-being of female older people than male. However, the result was contrary to the finding of some scholars (38). For individuals' political look, volunteer services did not significantly enhance the well-being of older people who were party members, while non-party members' well-being could be significantly improved. In terms of economic status, taking the median as the boundary, this paper classified older people whose economic status was higher than or equal to the median as good economic status, while the left was sorted as general economic status. The results showed that participation in volunteer services by well-off older people had a more significant effect on the well-being than those of the general economic status.

### Intermediary Mechanism Analysis

To further discuss whether older people's participation in volunteer service affected well-being by enhancing positive emotions and suppressing negative emotions, the mediating effect test was conducted and the results were shown in Table 7.

**Table 7:** Test results of mediating effect.

	**(5)**	**(6)**	**(7)**	**(8)**
	**Positive**	**Happiness**	**Negative**	**Happiness**
Volunteer	0.411***	0.149*	0.057	0.224***
	(0.103)	(0.056)	(0.156)	(0.056)
Positive		0.189***		
		(0.009)		
Negative				−0.102***
				(0.006)
Control variables	Yes	Yes	Yes	Yes
*N*	4,403	4,403	4,403	4,403

***, ** and *, respectively, indicate the significance of variables at the statistical level of 1, 5, and 10%.

From the results of models (5) and (6), it could be seen that older people's participation in volunteering could enhance their positive emotions, which in turn contributed to their well-being. The coefficient on the core explanatory variable became smaller and its significance decreased but remained significant at the 10% level, thus it could be argued that positive emotions played a partially mediating role. Based on models (7) and (8), we could know that there was a significant association between negative emotions and well-being when controlling for participation in volunteer services. However, the association between volunteer service and negative emotions was not significant, implying that volunteer service may not affect well-being through negative emotion changes. To ensure the accuracy of the result, this paper used the Bootstrap method to test the indirect effect. The results showed that the indirect effect coefficient was −0.004 (*p* = 0.688>0.1), which was not significant. It can thus be concluded that older people's participation in volunteering did not lead to changes in well-being by affecting negative emotions. Kahana et al. (26) suggested that this may be due to the fact that negative emotions were more associated with negative life events and circumstances and less associated with social engagement.

## Discussion

### Participation of Older People in Volunteer Services Can Improve Their Well-Being

Activity theory suggests that the higher the level of social participation, the higher the life satisfaction. The logic behind this is that life satisfaction stems from the clear self-understanding, which in turn stems from a new role, and a new role stems from social participation. Therefore, through participation in formal or informal volunteering, older people gradually develop a new role and a clearer understanding of themselves, which in turn increases their sense of well-being.

Many scholars have found that older people's participation in volunteer service has a positive impact on well-being (14–17). Willigen (13) pointed out from a life course perspective that the roles played by individuals had different meanings at different stages of life. For young people, the volunteer role may be an extension of an existing role, such as being a good parent, employee, etc. For older people, however, volunteering is a substitute choice, for example, by volunteer service instead of watching television at home. Therefore, the volunteer role has a greater significance for older people. Pilkington et al. (4) indicated that individuals who participated in volunteer services received more social support from family and friends, which in turn enhanced well-being. Through volunteering, older people can establish and develop new social connections and build and strengthen their social support system. In addition, existing social connections also provide emotional and instrumental social support to enable them to continue volunteering and further enhance their well-being.

### Participation in Volunteer Services Contributes More to the Well-Being of Older Female Than Older Male

This is contrary to the findings of Cao and Wang (38). One possible explanation is that after retirement, female shift from an instrumental role in the workplace to a purely emotional role, with a family focus and a shift in the focus of interpersonal interactions from colleagues to relatives, resulting in a compressed space of activities limited to the family internal domain, therefore, it is more prone to negative emotions (39). Participation of older female in volunteering is beneficial to their adaptation to the role change and expansion of social interaction channels.

### The Involvement of Non-party Members in Volunteer Services Can Significantly Improve Their Well-Being

For party members, Ding (40) pointed out that the *Constitution of the Communist Party of China* clearly stipulated the obligations of party members, requiring them to serve the people wholeheartedly, be self-sacrificing and make more contributions. This “altruistic spirit” has been implicitly seen by party members as their own part of the job. Xie (41) also suggested that political look was a special kind of cultural capital in China. For many older party members, they still demand themselves by putting others before themselves, serving the people, etc. From this perspective, party members' participation in volunteer services may be seen as a process of fulfilling their duties, rather than as a means of enhancing their well-being. However, for non-party members, there may be relatively few channels for their social participation. Thus, participation in volunteer services, as an important way of social participation, prompts non-party members to form new roles and deepen their self-understanding in the process of volunteer service, effectively enhancing well-being.

### The Better the Financial Situation of Older People, the More Significant the Effect of Volunteer Services on Well-Being

Hierarchical theory of needs states that individual needs can be categorized from low to high into five needs: physiological, safety, love and belonging, respect, and self-actualisation. Generally speaking, individuals will only pursue higher level needs when the lower level needs are satisfied. From this perspective, volunteer services can be seen as a channel for self-actualisation that can satisfy higher level needs. Therefore, for individuals with good economic status, after the lower level needs are satisfied, it is obvious that participating in volunteer services as a way to gain social connection and recognition, and to realize self-worth, has a more significant effect on their well-being. Conversely, for individuals of modest means, the more pressing needs are likely to be physiological and safety needs, and the pursuit of higher-level needs is not as high, thus volunteer services participation has a relatively small contribution to their well-being.

### Older People's Participation in Volunteer Services Enhances Well-Being by Influencing Positive Emotions

In China, the traditional virtue of helping others is deeply rooted in people's hearts. People are happier when they help others, which means that when you give a rose to someone, you get a reward. Volunteer services as a means of social participation, in the process of helping others, people can gain a sense of being valued and identified, their self-worth is realized and they realize their importance (27), which is conducive to a higher positive emotions and thus a higher sense of well-being. Greenfield and Marks (7) also suggested that the impact of volunteer services on subjective well-being was mainly through increased life satisfaction and positive emotions, and Kahana et al. (26) reached the same conclusion. Furthermore, in return for providing services and assistance, older people who participate in volunteer services will also gain other social support and connections, strengthening their social networks, which will contribute to maintaining their positive emotions and enhancing well-being.

## Conclusions

Based on 2013 CHARLS data, the empirical findings of this paper show that Chinese older people's participation in volunteer services contributes to their well-being, which is more pronounced in older people who are female, non-party, and in good financial situations. The impact of volunteer service on well-being is mainly achieved through the enhancement of positive emotions. This paper systematically reviews the worldwide literature on the relationship between older people's participation in volunteer services and well-being, enriching the social support theory account of well-being, providing a test of empirical evidence of social support theory in China, and complementing the mechanisms by which participation in volunteer service affects older people's well-being.

In conclusion, firstly, all relevant authorities in all countries should play an advocacy and support role to promote the participation of older people in volunteer services. On the one hand, media campaigns are needed to guide older people to change their traditional role of being cared for and to encourage them to actively use their strengths to participate in volunteering. On the other hand, the government should also provide relevant policies to support the development of community volunteering organizations and provide a platform for older people to participate in volunteering. Secondly, when organizing volunteer services for older people, volunteer projects and content should be enriched so that they can choose volunteer projects to participate in according to their own situation. For example, for the party members, community backbone cultivation programmes can be designed to bring into play their role as role models, which in turn can lead to the participation of other community members. Finally, communities and grassroots organizations can provide suitable volunteering models and channels for older people according to the actual situation. For example, the Time Bank Model, through the introduction of time credits, allows individuals to accumulate time credits of equal length when providing services to others, which can later be exchanged for goods or services. At the same time, they receive social support and this objectively promotes the formation of mutual social networks and support systems, improving the social welfare of older people and safeguarding the welfare of them.

## Data Availability Statement

Publicly available datasets were analyzed in this study. This data can be found at: http://charls.pku.edu.cn/pages/data/2018-charls-wave4/zh-cn.html.

## Author Contributions

H-lY and W-cZ conceived this research. H-lY, ZS, W-cZ, SZ, and L-lT were responsible for the methodology. S-qZ, SZ, and L-xY conducted software analyses. S-qZ, ZS, Y-wT, and Y-yW conducted necessary validations. W-cZ and J-hW conducted a formal analysis and managed the investigation. Y-dY, ZS, SZ, Y-wT, and W-cZ gathered resources, curated all data, wrote and prepared the original draft, and were responsible for project administration. LX, L-lT, and Z-yL reviewed and edited the manuscript, were responsible for visualization, supervised the project, and acquired funding. All authors contributed to the article and approved the submitted version.

## Funding

This work was supported by the Later funded projects of National Social Science Foundation (Grant number: 21FRKB003).

## Conflict of Interest

The authors declare that the research was conducted in the absence of any commercial or financial relationships that could be construed as a potential conflict of interest.

## Publisher's Note

All claims expressed in this article are solely those of the authors and do not necessarily represent those of their affiliated organizations, or those of the publisher, the editors and the reviewers. Any product that may be evaluated in this article, or claim that may be made by its manufacturer, is not guaranteed or endorsed by the publisher.
